# State of the Art *in Silico* Tools for the Study of Signaling Pathways in Cancer

**DOI:** 10.3390/ijms13066561

**Published:** 2012-05-29

**Authors:** Vanessa Medina Villaamil, Guadalupe Aparicio Gallego, Isabel Santamarina Cainzos, Manuel Valladares-Ayerbes, Luis M. Antón Aparicio

**Affiliations:** 1 Biomedical Research Institute (INIBIC), CHU A Coruña, Xubias de Abaixo s/n, PC 15006, A Coruña, Spain; E-Mails: lupe.aparicio@gmail.com (G.A.G.); Isabel.santamarina.cainzos@sergas.es (I.S.C.); manuel.valladares.ayerbes@sergas.es (M.V.-A.); 2 University of A Coruña (UDC), PC 15006, A Coruña, Spain; E-Mail: Luis.M.Anton.Aparicio@sergas.es; 3 Medical Oncology Department, CHU A Coruña, PC 15006, A Coruña, Spain

**Keywords:** bioinformatics, cancer, networks, pathways, systems biology

## Abstract

In the last several years, researchers have exhibited an intense interest in the evolutionarily conserved signaling pathways that have crucial roles during embryonic development. Interestingly, the malfunctioning of these signaling pathways leads to several human diseases, including cancer. The chemical and biophysical events that occur during cellular signaling, as well as the number of interactions within a signaling pathway, make these systems complex to study. *In silico* resources are tools used to aid the understanding of cellular signaling pathways. Systems approaches have provided a deeper knowledge of diverse biochemical processes, including individual metabolic pathways, signaling networks and genome-scale metabolic networks. In the future, these tools will be enormously valuable, if they continue to be developed in parallel with growing biological knowledge. In this study, an overview of the bioinformatics resources that are currently available for the analysis of biological networks is provided.

## 1. Introduction

The term “systems biology” has emerged recently to describe the frontier of cross-disciplinary research in biology. The suffix “Omics” encompasses a variety of new technologies that can help explain both normal and abnormal cell pathways, networks, and processes via the simultaneous monitoring of thousands of molecular components. Omics studies encompass many technologies, including genomics (the quantitative study of protein-coding genes, regulatory elements and noncoding sequences), transcriptomics (the quantitative study of RNA and gene expression) [1], proteomics (the quantitative study of protein abundance and protein modifications) [2], and metabolomics (the quantitative study of metabolites and metabolic networks) [3]. Omics studies also include research areas that have developed in the era of post-genomic biology and medicine, such as pharmacogenomics (the quantitative study of how genetics affects a host’s response to drugs) and physiomics (the quantitative study of physiological dynamics and the functions of whole organisms), as well as other fields, including nutrigenomics (a rapidly growing discipline that focuses on identifying the genetic factors that influence the body’s response to diet and how the bioactive constituents of food affect gene expression), phylogenomics (the analysis of genome data and evolutionary reconstructions, especially phylogenetics) and interactomics (the study of molecular interaction networks). The systems biology field overlaps with several emerging, post-genomic disciplines, such as synthetic biology [4], systems microbiology [5], systems biotechnology [6], integrative biology [7], systems biomedicine [8], and metagenomics [5]. Numerous definitions of systems biology have been proposed [9], but to date, no universally accepted definition has been accepted, which reflects the difficulty of encompassing a heterogeneous school of thought with a comprehensive yet concise definition. However, each of the proposed definitions revolves around a fundamental understanding of biological systems that is based on the underlying interactions of the components.

Recent experimental and theoretical work has begun to supply lacking biological details. In a small but growing number of systems [10,11], the examination of signaling events at the level of individual molecular interactions is possible. The theoretical and computational counterparts to these experimental findings are also now emerging [12]. These tools enable researchers to apply basic chemical principles to predict and analyze the functioning of molecular circuits at the single-molecule level.

The evasiveness of the gene concept has become fully apparent only in the last decade [13] with the analysis of sequenced genomes and extensive studies of the transcriptome with new techniques. Several facts highlight the complexity of the relationship between an organism’s phenotype and its genome [14]. As a result, in the past five years, the concept of the gene has been the subject of substantial revisions [13,15].

Enormous progress has been made in understanding critical cellular processes, such as cell cycle regulation, DNA repair, apoptosis, transcription, cell migration, and matrix structure, which are essential to the understanding and treatment of cancer [16–20]. However, cancer is not only a disease of cells but is also a disease of various systems and components that interact at both a molecular and cellular level to lead to the initiation and progression of the disease [21–24]. These interacting systems are intertwined by crosstalk between the genes in a cancer cell, the signal transduction pathways within a cancer cell, the cells in a tumor, the tumor and the microenvironment, and the individual and the macro-environment. Furthermore, the changing interactions of these systems in a dynamic environment underscore the inherent complexity of the disease. Until recently, a reductionist approach to cancer research has been made in order to understand each of these components. However, further integration across components or scales has been limited, primarily by a lack of the technology and tools needed to interrogate these systems at a higher level.

In the past 10 years, new technologies have been developed that have generated extensive genomic and proteomic data, as well as other types of genome-wide information [25,26]. Additional novel technologies have also made the imaging, isolation of rare cells, and organotypic culturing procedures possible. Together, these developments have facilitated an expansion of cancer research to include an integrative systems approach [27].

This review considers the chemical and physical types of complexity within cancer signaling pathways. Furthermore, the tools available to analyze signaling network data, which are accessible to specialized bioinformaticians and general research scientists, are detailed.

## 2. The Biochemical Networks in Cancer

**Figure 1:**
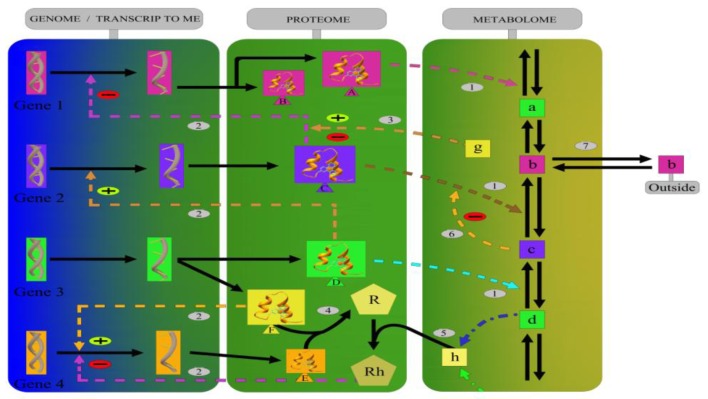
Biological networks represent various types of molecular interactions, including (1) enzyme catalysis, (2) the post-transcriptional control of gene expression by proteins, (3) the effect of metabolites on gene transcription mediated by a protein, (4) protein interactions, (5) the effect of a downstream metabolite on transcription, (6) feedback inhibition/activation of an enzyme by a downstream metabolite, and (7) the exchange of a metabolite outside of the system. The solid lines represent direct interactions, and the discontinuous lines represent possible interactions in tumor cells.

The full complexity and the multidimensional nature of biochemical networks that take into account the genome, transcriptome, proteome, and metabolome data highlight the intricacy and interdependency of these systems (Figure 1). Understanding how biological networks behave could help to explain the general mechanisms of tumor cell systems.

The types of networks that are currently available include, but are not limited to, protein interaction networks, genetic interaction networks, transcription factor-target regulation networks, miRNA-target regulation networks, kinase-substrate phosphorylation networks, and metabolic pathway networks.

Analogous to a sequence analysis, the development of a biological network has three similar stages: (1) the construction of the network by large-scale experimentation and computational predictions [28–30], (2) a pairwise network comparison to find the conserved edges as “interologs” or “regulogs” [31] with the building of general network alignment tools [31,32], and (3) an investigation of the conservation level and the evolutionary changes of the biological network.

Biological networks are characterized by their functional relationships, such as the binding, expression, regulation, and phosphorylation of proteins. Biological networks could be categorized as collaborative or regulatory networks. Collaborative networks are the biological networks with reversible edges (*i.e.*, either the edges are undirected or directed but reversible). In this study, the term reversibility means that a reversed edge is biologically possible between a pair of nodes. Regulatory networks have irreversible edges (*i.e.*, a reversed edge may not be biologically possible). By this definition, transcription factor-target regulation networks, miRNA-target regulation networks, and kinase-substrate phosphorylation networks fall into the regulatory network group and protein interaction networks, genetic interaction networks, and metabolic networks fall into the collaborative network group [33].

The transcriptional regulation of gene expression is carried out by transcription factors that bind to the transcription start site upstream of a gene. The recognition of a binding site is often specific to the DNA sequence [34]. The post-translational modification of a kinase’s substrate also involves the recognition of sequence patterns in the substrate’s phosphorylation site [35]. A protein’s function gradually changes as the protein’s sequence changes; however, most proteins do not change functions radically as their sequences are conserved. Metabolic enzyme networks are constructed using enzymes as nodes. The edges between two nodes are connected if the product of one node serves as the substrate of the other node. Metabolic reactions process chemical compounds into energy and nutrition, and most are essential for cellular survival.

Therefore, the ultimate goal of cancer systems biology research is to understand how individual molecule changes affect the function, organization and collaboration of cells. Certain factors may also influence and shape the landscape of tumor biological networks. The external environment has been shown to influence the conservation of regulatory relationships and network motifs in prokaryotic transcription factor-target networks [36]. Relationships tend to be conserved in organisms that live in similar environmental niches, despite large evolutionary distances. Whole-genome duplication events rapidly reorganize transcription regulation networks through the survival and functional divergence of the organisms with genome duplications [33]. In addition, the regulatory networks could affect the survival of organisms with duplicated genes by feedback mechanisms [37].

Genomic, proteomic, and metabolomic technologies have given researchers the tools to unravel additional underlying mechanisms that lead to cancer, which has resulted in an explosion of data. However, to yield significant insight, the systematic integration of data for cancer research requires a systems biology computational approach. This approach must combine the use of existing knowledge to account for what is known with the massive amounts of high-throughput data to determine what is not known. Biological knowledge is accounted for, using pathway models and mechanistic dynamic simulations of biological constituents (e.g., genes, proteins, and metabolites organized in pathways and networks). Unknown relationships are accounted for by data mining or reverse-engineering approaches that extract patterns and relationships from high-throughput data. Both of these approaches rely on the development of experimental methods that analyze the interaction, expression, and localization of biological molecules in a quantitative manner.

## 3. Bioinformatic Tools for Pathway Analysis

Along with the ability to generate a large amount of data per experiment, high-throughput technologies have also brought experimental challenges, such as translating large amounts of data into a better understanding of biological phenomena. Independent of the platform and the analysis methods used, in many cases, the results of a high-throughput experiment are lists of differentially expressed genes. Translating these lists into a better understanding of underlying biological phenomena is a common challenge faced by all researchers. In particular, placing high-throughput data into context within a whole organism remains a daunting challenge.

Currently, the overrepresentation approach [38] and the functional class scoring techniques [39], which are used to analyze high-throughput data, are limited by the fact that each functional category is analyzed independently without a unifying analysis at a pathway or system level. This approach is not well-suited for a systems biology approach that aims to account for system-level dependencies and interactions, as well as to identify perturbations and modifications at a pathway or organism level [40]. Several pathway databases, such as KEGG [41], BioCarta, and Reactome [42], currently describe metabolic pathways and gene signaling networks, thereby allowing a more complex and useful analysis in a pathological and non-pathological manner. All of the pathway analysis tools currently available use the overrepresentation approaches and fail to take advantage of the much richer data contained in these resources. GenMAPP/MAPPfinder [43,44] and Gene-Sifter use a standardized *Z*-score. PathwayProcessor [45], PathMAPA [46], Cytoscape [47], and PathwayMine [48] use Fisher’s exact test. MetaCore uses a hypergeometric model, while ArrayXPath [49] offers both Fisher’s exact test and a false discovery rate. VitaPad [50] and Pathway Studio [51] focus on visualization approaches alone and do not offer any detailed analyses. Finally, the impact analysis method, which is implemented as a Web-based tool, and Pathway-Express, which is freely available as part of the Onto-Tools software, use a systems biology approach to identify pathways that are significantly impacted by any condition that has been monitored by a high-throughput gene-expression analysis. The impact analysis method incorporates a classical probabilistic component along with important biological factors that are not captured by existing techniques, including the magnitude of the expression change of each gene, the position of the differentially expressed genes on the given pathways, the topology of the pathway that describes how these genes interact, and the type of signaling interactions between them.

**Figure 2:**
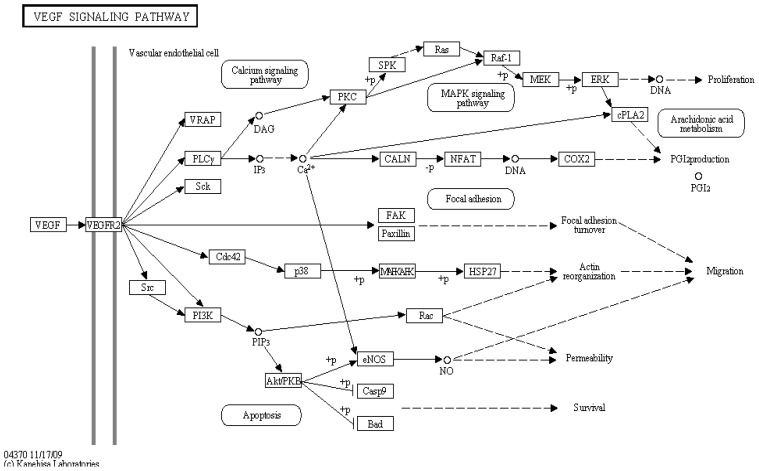
A map of the VEGF signaling pathway (as an example of an important target in cancer therapy) obtained from KEGG is shown [41]. The gene expression studies are used as an independent predictive method for the prognosis. In cancer genomics studies, tremendous effort has been devoted to pathway-based analysis. Pathway analysis is a promising tool to identify the mechanisms that underlie disease, the adaptive physiological compensatory responses and new avenues for investigation. Different pathways have different biological functions. Thus, studying each pathway separately is reasonable. Among the many pathways, only a few have been shown to have predictive power for the development of cancer. In this sense, KEGG could be a useful tool to identify genetic signatures [52].

Pathway-level analysis is a powerful approach that enables the interpretation of post-genomic data at a higher level than the individual biomolecules (Figure 2). The evidence of pathway dysregulation is combined, which allows for the identification of additional pathways with altered activities that would not be highlighted when the analysis is applied to any of the functional levels alone (*i.e.*, the genome, transcriptome, proteome or metabolome).

## 4. Bioinformatics Tools for Systems Biology

**Figure 3:**
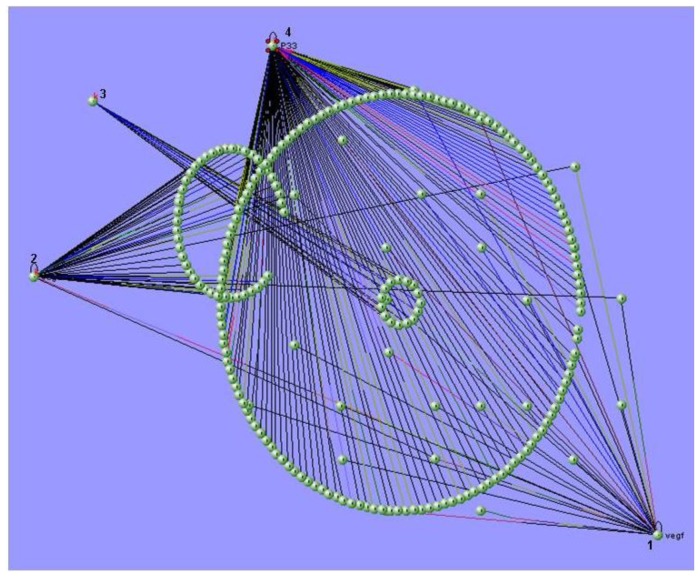
An illustration of the multi-scale visualization of biological interactions using VisANT’s metagraph capability. VisANT [54] is an integrative software platform for the visualization, mining, analysis and modeling of biological networks. This software extends the applications of GO for network visualization, analysis and inference. The image shows the biological networks between the molecules that belong to the VEGF signaling pathway. (1) VEGF, (2) VEGFR2, (3) COX2 and (4) p33.

The field of bioinformatics has blossomed in the last 10 years, and as a result, a large, increasing number of researchers are generating computational tools to solve problems relevant to biology. While many systems biology approaches involve mathematical and computational modeling, the development, maintenance, and dissemination of tools for systems biology are significant challenges. Examples of these challenges include the development of data repositories, data standards and software tools for the simulation, analysis and visualization of system components, such as biochemical networks (Figure 3). Another difficulty is the application of high-throughput molecular profiling technologies, which often require sophisticated data processing and analyses. These analyses also typically involve elements of signal processing and statistical analysis. As the resulting quantitative measurements are transferred to formal mathematical models for simulation purposes, the endeavor becomes more like systems biology and less like bioinformatics.

The practice of systems biology seeks to comprehend the complexity of organisms or organism subsystems by combining many different kinds of data, such as mRNA levels, protein levels, protein-protein interactions, protein-DNA interactions, protein modifications, and biochemistry data, to create predictive models [53]. The computational analysis of this data has become increasingly important, and many more tools and models for the interpretation of healthy and non-healthy biological data have been developed in recent years. However, not all of these methods are publicly available or permit bulk online submissions. Moreover, some tools, particularly the tools for individual organisms, require special training, and these tools may also be mutually interdependent.

**Figure 4:**
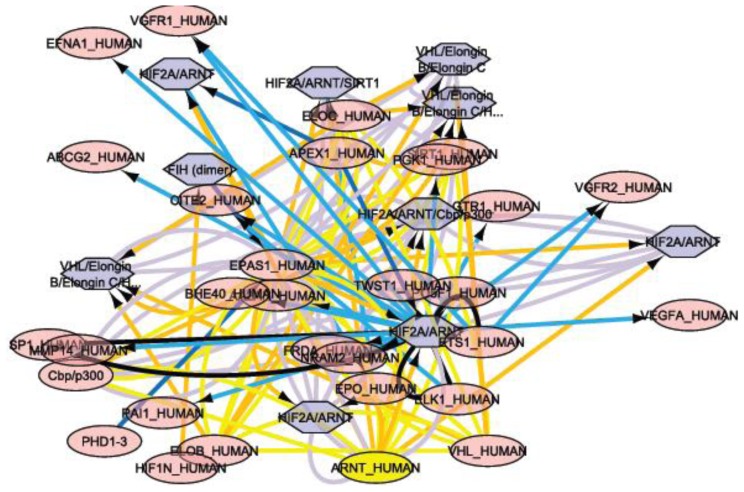
Cytoscape is an open-source bioinformatics software platform for visualizing molecular interaction networks and integrating these networks with other state data. Cytoscape was used to model the interaction networks for VEGF-A (also called vascular permeability factor (VPF). The software found 56 interaction networks at a pathway level, and one of these interaction networks, the HIF-2-α network, is shown with 40 nodes and 114 edges. The importance of this figure is to provide the reader understanding of the level of complexity to which we refer when we speak of interactions at the gene or protein level illustrating this by VEGF-A pathway. Each node represents a molecule that interacts with HIF-2-α; in this case 40 molecules interact with this important marker in different malignancies, through 114 edges.

As biological research accelerates through the development of new technologies and instrumentation, biological databases have become an indispensable part of scientific research. The construction and maintenance of primary databases, such as GenBank [55] and Protein Data Bank [56], have long been recognized as important bioinformatics work. Primary biological databases serve as repositories for experimentally derived information and are also the basis for the development of secondary databases that capture higher-level knowledge. An example of a secondary database is the Pfam database of proteins families and domains [57]. Concomitantly with the development of biochemical systems biology, the databases that capture the properties and processes of biochemical networks have emerged as an important niche of secondary biological databases. The ecosystem of these databases and the tools associated with these databases are rapidly growing and include metabolic pathway databases organized around the BioCyc project [58], a database of the human biological pathways [42], a database of the interactions between small molecules and proteins [59], and databases of protein-protein interactions [60]. As these databases attempt to reconstruct and organize information concerning the interactions between cellular components, they also attempt to build higher-level knowledge and theories concerning the biological processes they archive. This *in silico* knowledge is greatly needed because the integral complexity of most biological processes is beyond what is comprehensible to the human mind. Therefore, these “systems biology databases” often provide important foundations for the quantitative modeling of biological systems. In certain cases, these databases allow for the direct export of mathematical models. Additionally, the first collections of the mathematical models of biological processes have been developed (databases of models), which solely archive and curate the mathematical models in the Systems Biology Markup Language (SBML) for future use and refinement [61]. Tools for the visualization of network structures and the overlay of simulated and experimental data are greatly needed for systems biology research. These tools include the yEd graph editor for editing networks and tools for the visualization of “omics” data in the context of biochemical networks, such as Cytoscape [47] (Figure 4) and the Pathway Tools Omics Viewer [62].

The rationale for establishing a Working Group on Agents in Bioinformatics (BIOAGENTS) was to improve the field of bioinformatics by designing and implementing new, flexible information and communication technology tools. These tools should be able to support the analysis of biological data, partially distribute the computation burden of the large amounts of high-throughput data, and reduce the need to transfer large amounts of data. From this perspective, software agents can play a major role. The scope of the Working Group was to promote collaborations between software agents and the bioinformatics communities with the aim of creating synergies for the modeling of complex biological systems [63].

The combination of software agents with bioinformatics presents a two-fold opportunity. First, the domain of bioinformatics, which contains extensive and growing database resources and analysis tools, provides an appropriate domain for the application of agent technologies. Second, the combination of agents with bioinformatics allows for the deployment and testing of agent systems in a real-world setting with the possibility of making substantial contributions to human society. Additionally, a distinct and identified need exists for good solutions to improve the performance of existing bioinformatics systems, and agents may be able to contribute to this needed improvement. In this sense, there is a notably strong synergy between the two domains. This picture is both enhanced and complicated by the introduction of relevant infrastructure technologies that facilitate both bioinformatics and agent-based computing. For example, the Grid has become increasingly important to both communities and suggests a convergence to a service-oriented vision of bioinformatics underpinned by Grid-based virtual organizations. However, significant challenges still exist. Researchers from both communities generally require education in the other domain, and work must be undertaken to ensure that any solutions developed across both areas satisfy the needs of both domains. In many cases, the language of discourse is so distinct that the discussion of key issues becomes problematic. Additionally, the introduction of new technologies, such as the Grid, requires further efforts to understand and adopt the new technology, as well as improve the immaturity of fully deployed systems. Thus, the maturity at the interface is a key challenge.

## 5. The Virtual Cell

Computational systems biology is a rather new science [64]; however, its roots can be found in theoretical and mathematical biology. An example of these origins can be observed in the field of cell-cycle modeling. In the 1960s, mathematical models were proposed that attempted to explain key aspects of cell-cycle regulation from phenomenological observations. The field began to explode in the early 1990s when data were published on the underlying molecular regulatory network [65].

The cell cycle refers to a sequence of events that leads to the correct duplication of cells [66]. A complex regulatory network controls the proper order of the events within the cell cycle. The core controllers of this network in all eukaryotes are complexes of the Cdk and cyclin molecules. Various Cdk/cyclin pairs regulate the critical transitions of the cell cycle. These pairs initiate DNA replication at the transition from G1 to S-phase, and they play key roles in the induction of mitosis. In addition, Cdk/cyclin pairs inhibit the last steps of the cell cycle, which include the separation of the chromosomes at the end of mitosis and cell division. Key cell-cycle transitions are regulated by checkpoints, which ensure that cells start DNA synthesis only if adequate amounts of nutrients and growth factors are present, prevent mitosis from occurring until DNA replication is properly finished, and keep the chromosomes from separating unless the mitotic spindles are intact. If a problem arises in the cell cycle, the checkpoints signal to the core Cdk/cyclin modules to inhibit the further steps of the cell cycle [67].

Single-cell measurements and other new technologies have enabled the development of detailed, quantitative models of cell-cycle regulation. Mass spectrometry has provided data on protein level fluctuations during the cell cycle, identified members of important protein complexes, and revealed the phosphorylation states of the Cdk-regulated proteins [68]. Future targeted analyses of the key cell-cycle components will provide valuable data for biological modeling of the tumor cell cycle.

Several modeling platforms have been used in cell-cycle research [69]. These platforms usually guide the user through the process of model building to some type of data analysis. JigCell has been developed precisely for cell-cycle model simulations and data fitting [70]. This program can run multiple parameter sets to simulate various mutants, and it includes a comparator that tests how well the simulations fit the physiological details of the mutants. Although defining a suitable objective function for data that is not time dependent is difficult, JigCell provides tools for these estimations. Indeed, parameter optimization is one of the major challenges for modeling. High-throughput measurements rarely give reliable kinetic rates, and most often, these rates should be estimated from concentration profiles using a parameter optimization algorithm [71–73].

Searching for missing values is an example of the types of jobs that computational tools can undertake; missing values frequently pose problems in gene expression microarray experiments as they can hinder downstream analysis of the datasets. Although gene expression microarrays have developed much during the past years, the technology is still rather error prone, resulting in datasets with compromised accuracy and coverage. In particular, the existence of missing values due to various experimental factors still remains a frequent problem, especially in cDNA microarray experiments. Experimental data can also be used to infer undiscovered molecular interactions and propose the existence of protein regulation. Specific useful tools can handle this type of network data [74], and methods have been developed to search for missing interactions and infer network topology [75]. Because high-throughput data are available for the cell cycle of various organisms, researchers can start to think about how to fuse this high-throughput data to measurements on single gene perturbations to achieve a detailed understanding of the system. The computational identification of cell-cycle related transcription factors [76,77] is a promising initial result for these studies.

Another layer of complexity in cell-cycle models is the spatial distribution of regulatory molecules. Many crucial events happen in the nucleus, and many molecules are moved in or out of the nucleus during the cell cycle. Despite these localization limits, only a few cell-cycle models have considered the compartmentalization of the cell [78].

Recently, biological modeling has been enriched by new concepts that have helped to delineate cell-cycle models into sub-networks [79], find the exact timing of the cell-cycle transitions [80] and determine the irreversibility of these transitions.

**Figure 5:**
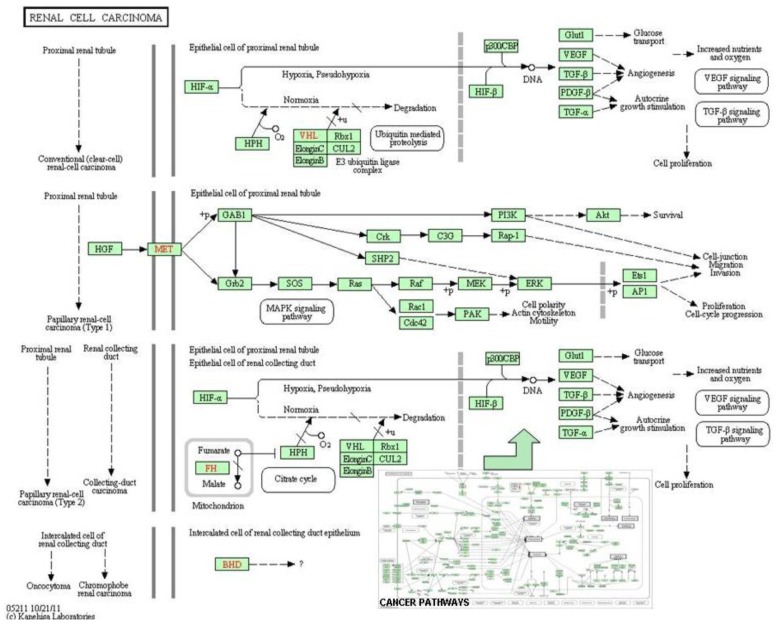
The cancer pathways obtained from KEGG are shown [41]. The complexity of the carcinogenic mechanisms leads to heterogeneity in the molecular phenotypes, pathology, and prognosis of cancers. Systems biology approaches leverage the signature genes as a representation of the changes in the signaling pathways, instead of interpreting the relevance between each gene and the resulting phenotype. At the bottom right side of the figure is represented the complex system of communication that exists in the signaling pathways in cancer (KEGG map05200). Within this complex of roads we can find among others, the signaling pathways that characterize kidney cancer (KEGG map05211).

**Figure 6:**
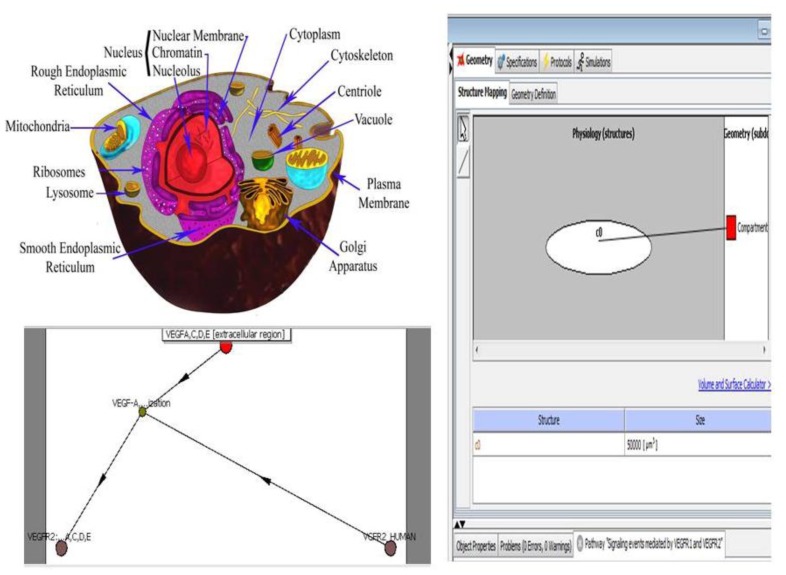
The challenge of understanding intracellular activity is being addressed by computational approaches, such as the Virtual Cell System. While this graphical representation of the VEGF network provides additional information not available by considering the individual pathways separately, it is still a vast simplification. The graphic is merely a static representation of several dynamic processes occurring concurrently with several intertwined feedback loops. The only way to effectively study the effect of either mutations or therapeutic interventions is to create a quantitative model of the network that integrates the dynamics of the individual pathways and their interconnections, which can be simulated on a computer. By representing aspects of an *in silico* cell, the model can then be used to explore a variety of questions.

Placing the core cell-cycle machinery into the larger context of cell physiology, deciphering how a cell copes with checkpoint problems, determining how a cell responds to environmental changes, and understanding why some cells leave the cell cycle to commit suicide remain major challenges for future work. Understanding these issues will lead us to the future goal of understanding how perturbations of the human cell cycle machinery lead to tumor formation. Indeed, the mathematical modeling of cancer development is another active research field [81–83]. Various ideas exist on how to model tissue growth computationally [84,85]. Predictive cell-cycle models embedded into complex tissue models can help us understand the dynamics of cancer formation (Figures 5 and 6).

The Virtual Cell [86] is a unique computational environment for the modeling and simulation of cell biology. This platform has been specifically designed to be used by a wide range of scientists from experimental cell biologists to theoretical biophysicists. The creation of biological or mathematical models can range from simple models used to evaluate hypotheses or interpret experimental data to complex multilayered models used to probe the predicted behavior of complex, highly nonlinear systems. These models can be based on both experimental data and purely theoretical assumptions.

The Virtual Cell has been deployed as a distributed application that is used over the internet. Users can build complex models with a web-based Java interface to specify compartmental topology, compartmental geometry, molecular characteristics, and relevant interaction parameters. The Virtual Cell automatically converts biological descriptions into a corresponding mathematical system of ordinary and/or partial differential equations. Distinct biological and mathematical frameworks are encompassed within a single graphical interface. A user, competent in mathematics, may directly specify the complete mathematical description of the model by bypassing the schematic interface. The Virtual Cell will then solve the equations by applying numerical solvers and generate appropriate software code to perform and analyze simulations. The results can be displayed and analyzed online or downloaded to the user’s computer in a variety of formats. The software is freely accessible to all members of the scientific community.

## 6. Systems Biology and Cancer

To understand how a cancer cell is functionally different from a normal cell, it is necessary to assess the changes in the dynamics of overall networks, rather than the changes in the individual pathways. As networks tend to be complex with extensive crosstalk between pathways and important feedback loops, representing networks in the form of a computational model that can be used for a rigorous analysis is crucial. As with most complex diseases, in the instance of cancer, an additional complication arises in that for many aspects of the diseases, several different scales must be integrated. Recognizing dynamic signatures can help improve the diagnosis of cancer, and an understanding of the mechanisms that lead to cancer can help with the design of sophisticated perturbations that can disrupt cancer progression.

Despite significant advances in the understanding of tumor biology, improvements in patient outcomes in terms of cure rates and survival times have not met many expectations. In the past 50 years, major advances have been made in scientific knowledge and technology. Additionally, increasing amounts of money are being spent to meet the clinical challenges of cancer. However, further progress is certainly needed.

Systems biology has emerged during the past decade as a powerful new paradigm for life-science research. This discipline is based on the premise that the properties of complex systems, which consist of many components that interact with each other in nonlinear, non-additive ways, cannot be understood solely by focusing on the components. The system as a whole has emergent properties that are not visible at the level of the individual parts. The availability of high-throughput data generation technologies, such as DNA microarrays, has made it possible to apply this paradigm in molecular biology and biomedicine. Large-scale transcriptional data allows the focus to shift from individual genes in linear pathways to large-scale networks of interacting genes. In many cases, the properties of the aggregate network, which possibly interact with the extracellular environment, are at the heart of pathological changes, such as cancer. Moreover, the system properties of interest are often embodied in its dynamics. For instance, changes in the structure of the network through mutations or epigenetic effects can lead to changes in the network dynamics that result in different physiological properties. In many cases, especially in the case of cancer, the phenomena at different scales are connected, such as the molecular- and tissue-level scales in tumor growth. The use of mathematical models is the natural framework for the study of the structure and dynamics of networks, which is perhaps the defining characteristic of the systems biology paradigm [87].

A key feature of cancer development is encapsulated in the interactions between the intracellular networks and the extracellular environment. Ultimately, a comprehensive understanding of cancer must link events at the molecular level with events at the tissue-level, organism level, and environmental level. These relationships can only be found with multi-scale mathematical models that integrate quantitative information at different scales, which is precisely the approach of systems biology.

To make progress in oncology research, more investigators and grant money have been added; however, these investments may not lead to more effective therapies. Further progress may require an entirely new approach, such as computational oncology. In the past, oncologists and oncology investigators viewed themselves as entirely separate from mathematicians and physicists. Bringing these once-disparate groups together to work on a common problem is long overdue and has spurred the development of the field of computational oncology.

**Figure 7:**
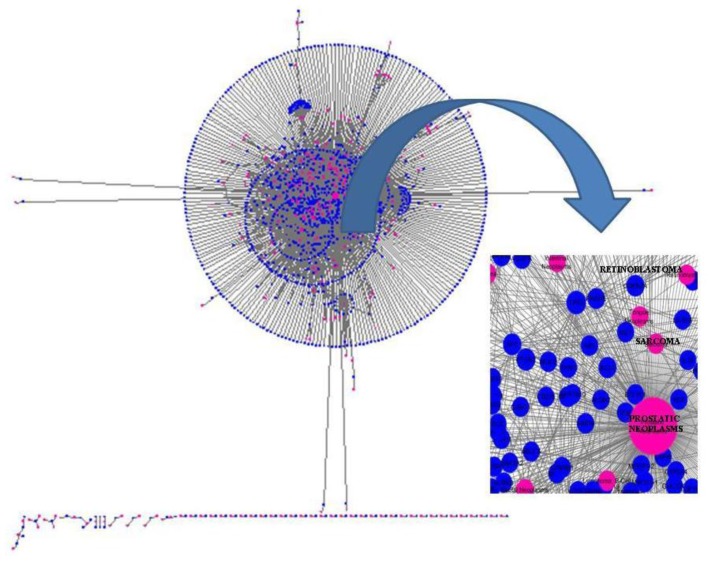
The ability to extract meaningful data from ever-expanding databases is an important area of development in computational oncology. Specifically, the relationship between genes and cancer is being documented by data-mining from large databases. In this figure, the interaction network for the markers involved in neoplasms is shown. DisGeNET [90] is a plugin for Cytoscape to query and analyze a network representation of human gene-disease databases. This figure illustrates the importance of addressing problems, such as which are the genes annotated to prostate, sarcoma or retinoblastoma neoplasms, for example, in expert-curated databases.

The study of systems biology requires the modeling of vastly complex components, which interact with each other on many levels. These models are crucial to the understanding of complex biological systems and are referred to as *in silico* models [88]. The dysfunction of any component of the network can lead to diseases, such as cancer, and the use of computational oncology techniques has allowed the development of sophisticated models to deepen our understanding of the origins of malignancies (Figure 7).

Using mathematical models to understand the behavior of cancer has generally proceeded in two directions: descriptive models and mechanistic models [89]. Descriptive models examine the gross characteristics of a tumor, such as its size, growth pattern and overall dynamics, and these models may seem the most intuitive to clinical oncologists. Mechanistic models focus on the various processes that lead to tumor growth in an attempt to understand the relative contributions of various components to overall tumor behavior. Both of these approaches are important for the further understanding of tumor biology. With data from *in vitro* experiments and animal models to develop and improve the mathematical models, the accuracy of the resulting models has been greatly improved.

Although current medicine employs single genes as molecular markers, thousands of markers analyzed simultaneously will probably be used as molecular marker profiles in the future. These molecular-oriented diagnostic techniques will be linked to the prediction and prevention of disease on a molecular level [29]. These developments are directly dependent on the future of computational oncology, which needs to combine large-scale and genome-wide data with biostatistical and bioinformatics analyses of model systems. This complex analytical process is the basis of a new scientific paradigm known as “integrative genomics”.

The use of systems biology for the study of cancer involves addressing the complex nature of the disease in the human body. Much experimental progress is being made to address these issues; however, the power of computation and computational modeling is still needed to help bridge this gap. The development of combined systems biology computational and experimental approaches to improve the understanding of human *in vivo* tumor behavior can greatly aid in improving pharmaceutical drug development and create a more rational, predictive approach to the application of therapeutic strategies.

## 7. Conclusions

High-throughput methodologies have allowed biological studies to move from a reductionist approach, such as the isolation of specific pathways and mechanisms, to a more integrative approach in which biological systems are seen as a network of interconnected components that provide specific outputs and functions in response to different stimuli.

The integration of known cancer genes into protein and signaling networks reveals the characteristics of these cancer genes within the networks. This approach shows that cancer genes often function as network hub proteins, which are involved in many cellular processes and form focal nodes for the exchange of information between many signaling pathways.

By providing a systematic and integrative framework for incorporating data and outputting predictions, systems biology has the potential to revolutionize the understanding of the mechanisms underlying cancer formation and to facilitate novel applications for cancer therapeutics.
